# Galactose-Induced Skin Aging: The Role of Oxidative Stress

**DOI:** 10.1155/2020/7145656

**Published:** 2020-06-17

**Authors:** Bauyrzhan Umbayev, Sholpan Askarova, Aigul Almabayeva, Timur Saliev, Abdul-Razak Masoud, Denis Bulanin

**Affiliations:** ^1^National Laboratory Astana, Nazarbayev University, Nur-Sultan, Kazakhstan; ^2^Astana Medical University, Nur-Sultan, Kazakhstan; ^3^S.D. Asfendiyarov Kazakh National Medical University, Almaty, Kazakhstan; ^4^Louisiana Tech University, Ruston, Louisiana, USA; ^5^School of Medicine, Nazarbayev University, Nur-Sultan, Kazakhstan

## Abstract

Skin aging has been associated with a higher dietary intake of carbohydrates, particularly glucose and galactose. In fact, the carbohydrates are capable of damaging the skin's vital components through nonenzymatic glycation, the covalent attachment of sugar to a protein, and subsequent production of advanced glycation end products (AGEs). This review is focused on the role of D-galactose in the development of skin aging and its relation to oxidative stress. The interest in this problem was dictated by recent findings that used *in vitro* and *in vivo* models. The review highlights the recent advances in the underlying molecular mechanisms of D-galactose-mediated cell senescence and cytotoxicity. We have also proposed the possible impact of galactosemia on skin aging and its clinical relevance. The understanding of molecular mechanisms of skin aging mediated by D-galactose can help dermatologists optimize methods for prevention and treatment of skin senescence and aging-related skin diseases.

## 1. Introduction

Skin aging is a complex process that depends on various extrinsic and intrinsic factors [1]. The most important intrinsic factors of aging include gender, ethnicity, and genetic variations [2]. A number studies on twins have shown a significant inherited component in skin aging [3–5]. Extrinsic factors can be divided into 3 main groups: (1) UV radiation and air pollution; (2) some diseases (e.g., diabetes); and (3) lifestyle choices, such as smoking, alcoholism and nutrition [6–8]. Solar radiation is the most crucial extrinsic factor capable of inducing premature skin aging and skin diseases in exposed areas of the body, e.g., the face and neck [9, 10]. Smoking and alcoholism can cause skin aging in nonexposed UV areas as well as accelerate aging caused by UV [11].

Among extrinsic factors, nutrition plays a vital role in the development of aging and aging-associated conditions [12]. In fact, an unbalanced diet with the domination of refined carbohydrates has been linked to the development of obesity and obesity-associated metabolic syndrome [13–15], which in turn is associated with diabetes and skin diseases [16], while a balanced nutritional diet helps maintain healthy skin and ensures its normal functioning [17–19]. The results of several studies have demonstrated that skin aging is also associated with a higher dietary intake of carbohydrates [20–22]. It has been established that the primary constructional molecules of the skin, elastin and collagen, can be damaged by carbohydrates via nonenzymatic glycation, the covalent attachment of sugar to a protein, and subsequent production of AGEs [8, 23–26], and these processes are closely linked to oxidative stress [27].

Glucose, fructose, and galactose are the essential simple sugars found in our diet. They could be consumed individually or in combination with each other in a form of more complex carbohydrates. The known mechanisms by which carbohydrates cause oxidative stress are the activation of mitochondrial oxidative metabolism of glucose, which leads to the generation of reactive oxygen species (ROS). In this case, ROS is generated through mitochondrial respiratory chain enzymes, xanthine oxidases, lipoxygenases, cyclooxygenases, nitric oxide synthases, and peroxidases [28–32]. The enhanced level of mitochondrial ROS leads to the activation of a number of biochemical pathways, such as the polyol pathway [33], the formation of AGEs [34–36], the activation of protein kinase C [37, 38], and the hexosamine pathway [39, 40], which in turn generate ROS [32]. Fructose-induced oxidative stress is also underlined by a similar mechanism [41].

There have been a number of debates about the critical role of high serum glucose levels as an “aging accelerator” for the skin [42]. This hypothesis has been supported by recent findings about diabetic and nondiabetic patients demonstrating that elevated levels of glucose can cause the fragmentation of the dermal connective tissue of the skin [8, 21, 42]. However, less attention is given to galactose, although there is data indicating that galactose (in particular, D-galactose or D-gal) is a more powerful glycation agent compared to glucose [43, 44] and is capable of inducing oxidative stress [45, 46].

Galactose is a C-4 epimer of glucose that combines with glucose to form the disaccharide lactose. There are two enantiomers of galactose: D- and L-galactose. In nature, the main form of galactose is D-gal. The major natural dietary source of galactose is milk and dairy products [47, 48]. Free galactose is also present in some fruits and vegetables, such as tomatoes, brussels sprouts, bananas, and apples [49]. In addition, the lactose hydrolysate syrup, as a sweetener, has been intensively used in biscuits, confectionery, and some dairy desserts containing high monosaccharide galactose content [48].

Galactose plays an important role in various physiological processes. For instance, it is involved in galactosylation of ceramide during myelin sheath synthesis of Schwann cells (PNS process) and synthesis of heparin/heparan sulfates [50]. It is known that galactose is formed endogenously in the human cells. A 70 kg adult male could synthesize up to 2 grams of galactose per day [51, 52]. In general, the possible reaction mechanism of endogenous galactose production is the lysosomal hydrolysis of galactose-containing glycoproteins, glycolipids, and proteoglycans [51, 52]. The level of galactose in the body can be elevated in two cases: (1) via increased consumption of foods rich in galactose, and (2) through metabolic disorders associated with genetic mutations in the enzymes of the Leloir pathway [53].

It was revealed that D-gal is able to trigger aging-like effects in experimental animals [54–57]. In fact, the use of D-gal for animal aging models has been intensively utilized for antiaging research worldwide since the early 1990s [58–60]. Numerous studies have been conducted to assess the aging mechanisms of the brain [61, 62] and heart [63] based on a D-gal animal model. Other reports demonstrated that D-gal could also be used for modeling liver [64] and kidney [65] aging. Although there have been a number of studies implicating D-gal-induced skin aging [66–86], the literature on this topic has been scanty and there is a need for these studies to be summarized and analyzed. In this regard, the current review is focused on the changes that occur during D-gal-induced skin aging. It also highlights the recent advances on the underlying molecular mechanisms of D-gal-mediated cell senescence and cytotoxicity.

## 2. Galactosemia: Etiology, Clinical Manifestations, and Treatment

**Figure 1:**
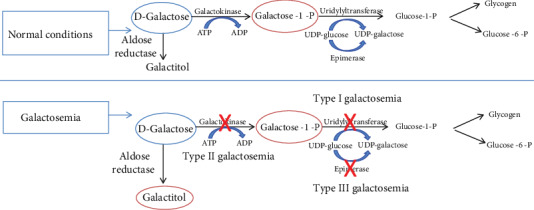
Metabolism of D-galactose.

Galactosemia is an inborn genetic metabolic disorder. It emerges as a result of the impaired processing of galactose, which can be subclassified into a few types [87]. It was revealed that the main pathway of galactose metabolism is the Leloir pathway which has several phases (Figure 1) [88].

The first key step is phosphorylation of D-gal by galactokinase (GALK1) to galactose-1-phosphate (Gal-1-P). Next, Gal-1-P is converted to UDP-galactose via D-galactose-1-phosphate uridylyltransferase (GALT) using UDP-glucose as the uridine diphosphate source. UDP-galactose 4′-epimerase (GALE) then converts the UDP-galactose to UDP-glucose. UDP-glucose returns into the pathway so that further galactose is converted into glucose-1-phosphate (G1P) and UDP-galactose. Finally, phosphoglucomutase converts the glucose-1-P into glucose-6-phosphate.

Classic galactosemia, also known as galactosemia type I, is a severe form of galactosemia that occurs due to the deficiency of GALT. Early manifestations of classic galactosemia are evident in the first few days after birth and initiation of breast-feeding. The most common symptoms are jaundice (74%), vomiting (4%), hepatomegaly (43%), failure to thrive (29%), poor feeding (23%), lethargy (16%), diarrhea (12%), and sepsis (10%). Another symptom that usually appears after two weeks as a result of galactitol deposition in the lens is cataract [89]. Later manifestations of the disease include growth delay, neurodevelopment impairments, liver and kidney dysfunctions, and premature mortality [89]. The acute symptoms of classic galactosemia can be resolved by an early implementation of galactose/lactose-restricted diet; however, the patients can still develop long-term complications such as neuropsychiatric impairments and dysfunctions of the ovaries [90].

Galactosemia type II was originally identified as a deficiency of GALK, while type III results from GALE deficiency [87]. The only consequence of GALK deficiency is the development and early onset of juvenile bilateral cataract; however, specific mechanisms underlying this localized-to-lenses effect remain unclear. Early diagnosis and treatment of *GALK* with a galactose-restricted diet may prevent or reverse the formation of cataracts [91].

GALE-deficient galactosemia is presented in three forms: generalized, peripheral, and intermediate. Most patients with GALE deficiency have poor activity of the enzyme in red blood cells (RBCs) and circulating white blood cells but normal or near normal in all other tissues. In the generalized form of the disease, GALE activity is profoundly decreased in all tissues while in intermediate, its form is characterized by deficient enzyme activity in RBCs and white blood cells and less than 50% of normal levels in other cells. Individuals with peripheral forms of GALE deficiency typically have normal growth and development, whereas patients who have generalized and intermediate forms show symptoms similar to classic galactosemia with different degrees of severity [90].

In addition to the 3 types of galactosemia described above, there may be other forms of this metabolic disorder. For example, the mild variant of classic galactosemia, also known as Duarte galactosemia, is characterized by partial GALT deficiency. This disease is more common than classical galactosemia (1 : 4000). Another common variant of galactosemia is the Los Angeles variant of galactosemia with AAC → GAC transition at nucleotide C, position 940 in exon 10, leading to an asparagine to aspartate substitution at residue 314 of human GALT enzyme (p.N314D) [92]. It was established that the frequency of p.N314D allele is approximately 11% in European populations and 8.3% for panethnic frequency [92, 93]. Therefore, in this case, the reduction of the GALT enzyme activity is more common and less diagnosed. Given the possibility of high galactose intake with a modern diet coupled with the presence of common variants of galactosemia, an analysis of the possible health effects of elevated galactose levels is necessary.

Although skin abnormalities have not been identified as clinical manifestations of galactosemia, irregular glycosylation of collagen was detected in bones of galactosemic patients [94]. Moreover, increasing experimental evidence, which we discuss in the subsequent sections, suggests that high D-gal concentrations can induce dermal cytotoxicity and aging-like skin changes. Thus, more studies are needed to address skin senescence and senescence-associated skin diseases in patients with galactosemia.

## 3. Dermal Toxicity of D-Galactose Studied *In Vivo*

We identified 21 publications where D-galactose-induced skin aging in rodents was studied [66–80, 82–86] (Table 1).

In 76% of these studies, the researchers mainly employed mice (16 studies), and only 24% used rats (5 studies). The most widely utilized mouse and rat strains were Kunming mouse strain (9 publications) and Wistar rats (3 publications). Eleven publications (52%) used male animals, 5 (24%) female animals, and 2 (10%) both genders, and 3 publications (14%) did not report gender. D-gal doses ranged from 50 to 1000 mg/kg, where D-gal at a dosage of 1000 mg/kg weight was the most often used in the experiments (9 publications). The age of animals varied from 4-6 weeks up to 22 months. The period of exposure was in the range of 30 days to 12 weeks.

These studies showed that significant changes in skin morphology occurred in rodents as a result of D-gal treatment. In particular, the data showed that the administration of D-gal in mice and rats caused skin thinning and worsening of fur quality [67, 68, 70, 71, 74, 76–79, 82–85]. Also, hair color changes and unique skin appearance with wrinkles and furrows were detected [80, 84] as well as the destruction of hair follicles in the skin [69, 84]. Tian and colleagues reported the apparent accumulation of subcutaneous fat and fewer cell layers in the skin of Kunming mice treated with D-gal [71]. Wang and coauthors showed that skin tissue angiogenesis was reduced after D-gal administration [76]. The skin moisture level, a known skin aging biomarker, was also decreased in the skin of D-gal-treated animals [70, 74, 80]. In addition, it was reported that the molecular biomarkers of skin aging such as p16 and p21 protein expressions [83, 95] were increased, and conversely, the level of sirtuin 1 (Sirt1) and cyclin D1 was reduced in the skin of animals treated by D-gal [74]. However, there are also contradictory results demonstrating that the administration of D-gal at a dose of 125 mg/kg/day, for 6 weeks, did not change skin water content in male Wistar rats [79].

In majority of the abovementioned studies, special attention was given to the quality of collagen fibers and collagen content [66–75, 78–80, 84, 86]. It was established that D-gal treatment reduced the total skin collagen in both mice and rats [66–75, 77–80, 84]: the type I collagen expression was downregulated [68, 79], while the number of type III collagen fibers was increased [68]. The data presented in these studies indicate that significant collagen fiber shortening and disordering occur in D-gal-treated animals [68]. Also, the dermal collagen fibers were sparse, slender, or broken [79]. Disorganization of collagen was noted in a loosely connected network in the skin of rats treated by D-gal [84]. As indicated by Ye et al., a loss of elastin was also observed in the skin of D-gal-treated animals [70]. In addition, the elastic fibers of the skin were reduced, thinned, and scattered in SD rats [82].

Dogs have also been used in tests for assessing D-gal-induced toxicity [96–104]. The main focus of research on dogs has been focused to assess the effects of galactose on eye damage [97–104]. Several studies have revealed that a 30% galactose diet induces galactosemia and development of diabetes-like microvascular lesions of the retina [97–101, 103]. It has also been shown that a diet containing 30% galactose rapidly accelerates cataract formation in galactose-fed dogs [102, 104]. Evidence shows that eye damage in galactose-fed dogs causes osmotic shock and is linked to aldose reductase [102]. There was only one study on a D-gal-induced aging model in dogs carried out by Ji and coauthors [96]. They demonstrated that D-gal, at 50 mg/kg daily subcutaneous injections for 90 days, increased the levels of MDA and suppression of SOD and GSH-Px in serum and brain tissue. It was reported that histopathological features were identified in the liver, kidney, heart, lung, spleen, and brain of galactose beagles. Moreover, a decreased expression level of proliferating cell nuclear antigen (PCNA) and enhanced expression levels of p16 and p21 were revealed in the D-gal-induced aging group compared with the young control group [96]. In contrast to studies conducted on rodents, assessment of the skin condition in dogs with experimental galactosemia was not carried out.

Thus, based on the presented data, it is reasonable to conclude that D-galactose induces skin aging in mammals. However, most of the studies were focused on the evaluation of antioxidant properties of new drugs or methods rather than exploring toxic effects of D-gal. To date, we know very little about effective dosages for skin aging induction since there is heterogeneity and inconsistency found in the above-cited publications. The proposed models have good experimental potential; yet, more studies are needed for the optimization of the laboratory animal skin aging models. The exact molecular mechanisms underlying D-gal-induced skin aging still remain unclear and require further investigations. In this regard, we discuss several pathways implicated in the D-gal-mediated skin cell senescence and cytotoxicity in the section that follows.

## 4. Molecular Mechanisms of D-gal-Induced Skin Aging

There is a growing body of evidence suggesting that D-gal is able to induce senescence of dermal fibroblasts *in vitro* [53]. For example, Elzi and coauthors showed that GALK1-deficient fibroblasts had increased senescence, which was associated with the accumulation of intracellular D-gal [105]. D-gal administration (10 mM of D-gal for 48 h) induced acceleration of senescence of GALK1-deficient fibroblasts while recovery of wild-type GALK1 expression led to the reversing of this process [105]. Another study showed that treatment of human dermal fibroblasts with D-gal (10 mg/mL) for two days resulted in 50% SA-*β*-gal-positive staining of cells and this was linked to the reduction of cell growth and G0/G1 phase arrest [106]. The notion that D-gal is able to affect the cell cycle in human fibroblasts has also been confirmed by Cui et al. [107]. The authors reported that the proportion of the cells in the G0-G1 stage was increased while the levels of G2-M cells and cell growth rate were decreased in the fibroblasts treated with 8 g/L of D-gal compared to the untreated cells. In addition, it has been established that D-gal affects genome integrity [108]. In this study, the incubation of porcine fibroblasts with D-gal (50 g/L) for 96 hours caused deformation of cell and chromatin shapes, induced chromatin condensation, and, in some cases, caused nuclear fragmentations and folds.

The most feasible mechanisms of D-gal-induced senescence are oxidative stress and suppression of the antioxidant system [109]. It has been demonstrated that the production of H_2_O_2_ significantly increased in mouse skin after D-gal treatment [84]. In turn, increased oxidative stress leads to lipid peroxidation, as evidenced by an increase in the concentration of MDA in the skin [72–74, 76, 78, 82–84]. In contrast, the activity of SOD was decreased in the skin after D-gal treatment [70, 73, 76, 78, 83, 84]. Some contradictory results have been reported about the activity of other elements of the antioxidant system. For instance, biochemical analysis of the D-gal-treated skin showed that activities of CAT, GSH-Px, and GSH were reduced in some studies [73, 82–84]. On the contrary, it was demonstrated that CAT [74] and GSH-Px activities were increased in D-gal-treated animals [72, 74].

A recent study based on untargeted metabolomic approach with mass spectrometry and dual liquid chromatography tested 14 main pathways, including the multiple redox, amino acid, and mitochondrial pathways, to understand the pathophysiology of long-term outcomes in classic galactosemia [110]. The findings of the study indicated that cysteine, vitamins E/B_3_, amino acid metabolism, and pathways involving mitochondria (e.g., carnitine shuttle, porphyrin metabolism, and nicotinate (niacin) metabolism) were impaired in plasma samples of the patients with classic galactosemia. Authors have suggested that galactosemia was associated with oxidative stress and/or perturbed redox signaling. It was also shown that at a pathologically high concentration (greater than 5.0 mM), D-gal increases lipid peroxidation, decreases total sulfhydryl content, and alters antioxidant defenses in rat plasma and erythrocytes [45]. Moreover, increased expression of biomarkers of the oxidative stress and decreased antioxidant defense were induced by D-gal in human fibroblasts *in vitro* [111, 112].

The important factors leading to oxidative stress are mitochondrial oxidative impairment and nicotinamide adenine dinucleotide phosphate oxidase (NADPH) oxidase activation resulting in overproduction of reactive oxygen species (ROS) [65, 113–116]. It was reported that D-gal reduced the efficiency of oxidative phosphorylation (OXPHOS) via declined transmembrane potential, decreasing ATP production and changing of respiratory function [65, 113, 114]. Upon galactose exposure, human fibroblasts tended to reduce the mitochondrial quantity and mitochondrial DNA (mtDNA) copy number and enhance mtDNA damage [111, 117, 118].

D-gal-induced oxidative stress probably depends on several factors. One of them may be the accumulation of AGEs [66, 119]. This is highlighted by a significant increase in AGE levels in the nude mouse skin treated by D-gal (1000 mg/kg) for 8 weeks [76]. In turn, AGEs can trigger cell damage through 3 main mechanisms: (1) accumulation of the AGEs in the extracellular matrix (ECM) (collagen and elastic fibers) initiates a crosslinking process between AGEs and ECM, which results in the reduction of connective tissue elasticity; (2) glycated modifications of intracellular proteins lead to an impaired cellular function; and (3) binding of AGEs to the receptor for AGE (RAGE) causes activation of inflammatory signaling pathways, NADPH oxidase activation, ROS generation, and apoptosis [120, 121]. Antibodies against RAGE and RAGE inhibitors were capable of inhibiting the generation of ROS from NADPH oxidase and suppressing the expression of proinflammatory cytokines *in vitro* [122, 123].

It has been reported that the ubiquitin-proteasome system (UPS) and autophagy are involved in the removal of AGEs [124]. However, the abovementioned factors themselves can have negative influence on the protein degradation systems in cells [125]. It was established that D-gal can disturb ubiquitin-proteasome system [125] and thus enhance the levels of glycated proteins in the cells. It should also be noted that autophagic flux alterations were detected recently in D-gal-treated skin fibroblasts [111, 117]. This can provide an insight on the additional mechanism of the acceleration of D-gal-induced cell senescence.

Oxidative DNA damage induced by D-gal may be crucial for the development of skin aging. The role of both nuclear and mtDNA damage in skin cells induced by excessive ROS is well described in relation to the UV-induced skin aging process [9, 126]. Similar DNA damage was found in fibroblasts treated with D-gal [111, 117, 118]. It was established that oxidative DNA damage activates the downstream mitogen-activated protein kinases (MAPKs) including extracellular signal-regulated kinase (ERK), p38, and c-Jun NH2-terminal kinase (JNK) [127]. These kinases act as upstream activators of nuclear factor-*κ*B (NF-*κ*B) and transcription factor activator protein-1 (AP-1). In their active states, both transcription factors function as repressors of collagen production and activators of matrix metalloproteinases (MMPs) in fibroblasts [128]. One of the MMP that is activated by NF-*κ*B and AP-1 is MMP-1. This metalloprotease is responsible for the primary step of collagen fiber fragmentation. Later on, two additional metalloproteases, MMP-3 and MMP-9, activated in response to the NF-*κ*B and AP-1, further degrade the collagen in the skin [129, 130]. This was consistent with the data on the enhanced expression of NF-*κ*B and MMP-9 in fibroblasts treated by D-gal as reported by Voets et al. [112]. In addition, NF-*κ*B activation was detected in various tissues of dogs after exposure to D-gal [96].

In dermal fibroblasts, the AP-1 transcription factor (described above) also acts as a repressor of the transforming growth factor beta (TGF-*β*) signaling pathway. This pathway is one of the main activators of collagen expression and expression of other ECM proteins in the skin [131]. Therefore, the downregulation of the TGF-*β* signaling pathway in response to ROS leads to the reduction of collagen expression and an increased rate of collagen degradation. In summary, activation of MMPs and repression TGF-*β* signaling have a significant negative impact on collagen production and skin aging. Moreover, it was indicated that D-gal reduced TGF-*β*-induced myofibroblast differentiation and collagen production [132].

ROS formation also induces low-grade inflammation, known as inflamm-aging, and is considered another critical factor of skin aging [133]. According to a recently proposed model, oxidative stress leads to accumulation of oxidized lipids and damaged epidermal cells. This triggers the complement system activation and inflammation, which in turn induces macrophage infiltration into the skin. Macrophages can release MMPs, but when overburdened with oxidized lipids and other toxic compounds, they tend to release ROS and proinflammatory cytokines [134]. In this context, it should be mentioned that the increased levels of expression of interleukin- (IL-) 1B and tumor necrosis factor (TNF) in fibroblasts treated by D-gal were detected [112].

Dermis vasculature is yet another important component that plays a crucial role in the skin aging process. During this process, the number and the size of vascular vessels significantly reduce, aiding to overall vasculature reduction in the dermis [135]. This reduction is suggested to be a result of vascular endothelial growth factor (VEGF) signaling cascade impairment [136]. However, the molecular mechanisms describing the galactose effect on the VEGF signaling pathway during the skin aging process are yet to be determined. For instance, Chen and coauthors demonstrated that D-gal induces senescence of human umbilical vein endothelial cells (HUVECs) and represses angiogenesis and wound healing *in vivo* [83]. On the other hand, treatment by D-gal did not significantly change VEGF production in retinal cells *in vitro* [137]. Some information exists for the diabetic, hyperglycemic conditions. Hyperglycemia also induces the generation of ROS, which impairs wound healing [138]. During wound healing, keratinocytes, fibroblasts, endothelial cells, macrophages, and platelets produce various growth factors and cytokines, one of which is VEGF [139]. Therefore, the impairment of VEGF may reduce the overall reepithelization process in the skin.

Taking together the information above, it is tempting to speculate that ROS produced via D-galactose accumulation may streamline through the same downstream effectors of ROS, such as transcription factors NF-*κ*B and AP-1, as well as the impairment of VEGF signaling, and hence further enhance the negative impact on collagen degradation and skin aging.

The possible role of galactose in the accumulation of metabolites in tissues, which can lead to cell damage, should also be taken into account. One of the possible underlying mechanisms of D-gal-induced senescence is osmotic shock caused by the accumulation of galactitol [63]. D-gal is usually metabolized by conversion to G1P via the Leloir pathway [88]. Intake of high doses of D-gal may also lead to galactitol accumulation via a reaction catalyzed by aldose reductase [66]. It was reported that human skin fibroblasts accumulated 20 times more galactitol than sorbitol when incubated in the presence of an unusually high concentration of galactose [140]. However, Kubo and colleagues suggested that the significance of osmotic shock for D-gal toxicity was exaggerated and this mechanism is based on free radical production [141]. They demonstrated that an abundance of galactitol leads to activation of aldose reductase, consequent exhaustion of the NADPH, and suppression of glutathione reductase activity. Also, it can promote changes in the oxidative status of the cells.

Furthermore, many studies demonstrated the possible key role of Gal-1-P in the development of toxic effects caused by an increased concentration of galactose in the blood [53]. Gal-1-P is the product of the first step of galactose metabolism, where galactose is converted into Gal-1-P by GALK1 [50]. In case of metabolic dysfunction or a very high level of galactose, it was established that Gal-1-P accumulates in different tissues [142], including skin fibroblasts [143] of galactosemic patients exposed to galactose. Recent studies have shown that the treatment of neonate skin fibroblast cultures with Gal-1-P significantly enhanced cellular levels of NO and inducible nitric oxide (iNOS) more than galactose treatment [144]. In addition, Gal-1-P downregulates expression of insulin-like growth factor 1 (IGF-1) [144]. Another study showed that elevated levels of Gal-1-P disrupt the phosphatidylinositol bisphosphate- (PI(P)2-) dependent signaling pathway in GALT-deficient tissues by restriction of the inositol phosphate turnover and reduction of the inositol level in tissues [145, 146]. In this context, it should be noted that inositol has antioxidant properties and reduces oxidative stress [147].

**Figure 2:**
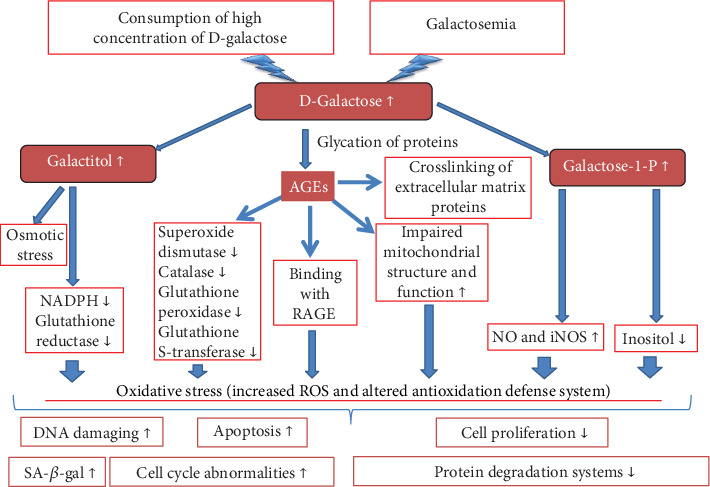
Schematic illustration of D-galactose cytotoxicity

In summary, galactose possesses a cytotoxic potential, and it is able to cause senescence of skin cells *in vitro*. The mechanisms underlying this process are not fully understood yet, but the most likely candidate for these mechanisms is the development of oxidative stress, which can also occur as an outcome of other toxic mechanisms. The main mechanisms of D-gal cytotoxicity are summarized in Figure 2.

## 5. Natural Antioxidants and Polyphenols in Studies on D-gal-Induced Aging

Aging models based on the administration of D-gal have been intensively utilized for validation of the antiaging efficacy of natural antioxidant compounds over the last decades [148]. In fact, the antioxidant properties of polyphenols have been tested in a number of studies using a model of skin aging based on the use of D-gal [149–152]. For instance, it was established that the combined treatment using resveratrol and calorie restriction restores hair condition, skin elasticity, and skin thickness in rats treated by D-gal [85]. In another study, apigenin (4,5,7-trihydroxyflavone), a flavone subclass of flavonoid, was able to significantly repair both collagen type I and type III density and thickness of the skin of D-gal-treated mice [67]. Ye and coworkers showed that extracts from *Idesia polycarpa*-defatted fruit residue containing phenolic and flavonoid components could improve the skin conditions of D-gal-treated mice (increase SOD activity; maintain collagen, elastin, and moisture content; and decrease MDA content) [70]. Tian et al. reported that persimmon-condensed tannin could dose-dependently reverse mouse skin aging induced by D-gal [71]. The same study established that another polyphenol, proanthocyanidin from grape seeds, possesses an antiaging activity for the skin but to a lesser extent than tannin [71].

Epigallocatechin gallate (EGCG), tea catechin, is one of the well-studied polyphenols up to date, with proven anti-inflammatory, antioxidant, and antiaging properties [153–156]. Chen and coworkers scrutinized the antiaging potential of EGCG on a D-gal-induced aging animal model [84]. The results showed that the conditions of the skin of EGCG-treated groups were improved such that the whole structure of the skin was better compared to the control. The levels of oxidative stress and the expression of EGFR proteins were significantly higher than those in the control group. These findings suggest that EGCG can effectively alter skin aging.

The antiaging effect of *Inula britannica* flower flavonoids on D-gal-induced aging mice was demonstrated by Chen et al. [74]. The results showed that *Inula britannica* could effectively improve the antioxidant enzyme activity of the aging mice, enhance the activities of SOD, CAT, and GSH-Px of skin tissue, and decrease the MDA content. In addition, it was also revealed that these flavonoids can help maintain the skin collagen, HYP, dermal thickness, and moisture content. Moreover, *Inula britannica* was able to decrease the number of cells arrested in the G0/G1 phase and increase the expression of Sirt1 and cyclin D1 along with a decrease in the expression of p16 and p21. These results indicate that *Inula britannica* extracts can be used as a potential natural antiskin aging agent.

In another study, Sukoyan et al. evaluated the effect of 2% *Cynara scolymus* L. (extracts of artichoke plant) on inflammation in a D-gal-induced skin aging animal model [80]. The data of the study showed that *Cynara scolymus* L. extracts were able to restore skin relative weight and hexosamine/collagen (HYP) ratio along with decreasing the activity of NF-*κ*B. Topical treatment also improved collagen metabolism and attenuated the progression of inflammation in a D-gal-induced skin aging model. In addition, Sulistyoningrum and coauthors demonstrated that dermal fibroblast count, density of dermal collagen, and plasma MDA were restored by *Muntingia calabura* aqueous leaf extract (MCALE) (35 mg/kg) and vitamin C (28 mg/kg) on a D-gal-induced mouse model of skin aging [86].

Apart from polyphenols, other natural antioxidant compounds have also been intensively studied with regard to their use for D-gal-induced aging research. Li and coauthors demonstrated that purified fractions of soluble polysaccharides derived from *Agaricus bisporus* (400 mg/kg) and vitamin C (200 mg/kg) had potential antiaging effects against D-gal-induced skin aging [69]. Moreover, Jing et al. showed that the acidic- and alkalic-extractable mycelium polysaccharides (extracted from *Agrocybe aegerita*) increased the amount of collagen in the skin in a D-gal-induced skin aging animal model [75].

Zheng et al. investigated antioxidant activities of marine pepsin-soluble collagen (PSC) derived from the skin of yellow goosefish (*Lophius litulon*) on oxidative stress in a D-galactose-induced skin aging animal model. It was established that PSC could decelerate the progress of wrinkle development and skin elasticity reduction and inhibit oxidative stress induced by D-gal [73]. The antiaging activity of low molecular weight peptide (LMWP) extracted from *Paphia undulate* was demonstrated by Chen et al. [82]. The researchers established that LMWP was able to reduce oxidative stress and enhance thickness and elasticity of the skin in a D-gal-induced animal model.

In addition to natural compounds, synthetic molecules have also been explored for their antiaging properties. Intradermal microinjection of dimethylaminoethanol (DMAE), an analog of the B vitamin choline and a precursor of acetylcholine, and compound amino acid (AA) caused an increase in dermal thickness, total collagen content, and collagen type I in D-gal-treated rats [79]. Another study demonstrated that argireline, a synthetic peptide, which is patterned from the N-terminal end of SNAP-25 protein, possesses a significant antiwrinkle activity, and it can improve morphology of the skin. Moreover, it can inhibit oxidative processes induced by D-gal [68].

Alternatively, stem cell therapy has been also considered a treatment of aging-associated diseases. For example, Zhang et al. demonstrated that the transplantation of adipose-derived stem cells (ADSCs) into the skin of D-gal-treated mice significantly improved skin elasticity and dermal thickness and accelerated angiogenesis [78]. Transplantation of ADSC reduced MDA and activated SOD activity, and it was associated with a decrease in mouse AGE levels post-ADSC treatment. Similar results were reported by Wang et al. [76]. In another study, Li and colleagues examined the combined treatment of adipose-derived stem cells (ADSCs) and fullerenol (as antioxidant) on skin aging induced by D-gal. It was found out that fullerenol suppressed the retention rate of transplanted ADSCs and enhanced dermal thickness and collagen ratio in mice [77]. Recently, Chen et al. investigated the effects of human embryonic stem cell-derived exosomes (ESC-Exos) on the aged mouse skin pressure ulcer model based on the administration of D-gal [83]. It was established that ESC-Exos promoted wound closure and increased angiogenesis. The senescence of vascular endothelial cells was significantly reduced after ESC-Exos exposure in D-gal-treated mice [83].

Taking the abovementioned into consideration, it could be concluded that natural compounds with antioxidant properties demonstrated significant potential in antiaging research, particularly for a D-gal-induced skin aging model.

## 6. Conclusions

This review discussed the evidence of D-gal-induced skin aging based on a range of *in vitro* and *in vivo* studies. It was shown that the toxic impact of galactose is manifested at high concentrations in the body [53]. However, to date, organ-specific systemic toxicity studies that have been conducted, particularly with regard to the skin during galactosemia or increased consumption of galactose, do not reflect the full picture of galactose toxicity. Also, little is known about the exact underlying pathogenic mechanisms. It can be assumed that D-gal induces skin aging in two ways: the direct impact of D-gal on skin cells and/or the indirect effect associated with the accumulation of toxic metabolites in the skin. Our understanding at this age leads us to suggest that cytotoxicity of D-gal is based on activated oxidative stress, as confirmed by the *in vitro* [65, 111–118, 144] and *in vivo* experiments [70, 72–74, 76, 78, 82–85].

Although a significant body of evidence suggests that high D-gal concentrations can induce skin aging, these are largely circumstantial [66–80, 82–86]. To the best of our knowledge, skin aging has not been identified in patients with galactosemia [87, 157]. On the other hand, abnormal glycosylation of collagen was detected in bones of galactosemic patients [94]. Based on this evidence, one should potentially expect to find abnormal glycosylated collagen in skin tissues in patients diagnosed with galactosemia. To address this point and validate our assumption, further research attempts are required along with the search for alternative underlying molecular mechanisms.

## Figures and Tables

**Table 1:** Skin changes induced by D-galactose.

	Strain	Gender	D-gal doses	Period of exposure	Age	Aging effects	Ref.
Mice							
1	C57BL/6J	Female	50 mg/kg daily subcutaneous injection	8 weeks	5 months	Skin HYP ↑	[66]
2	C57BL/6J	Female	1000 mg/kg daily subcutaneous injection	8 weeks	6 weeks	Dermal thickness ↓ Type I collagen fibers ↓ Type III collagen ↓	[67]
3	C57BL/6J	Male	1000 mg/kg daily subcutaneous injection	8 weeks	8 weeks	Dermal thickness, density ↓ p16 ↑ MDA ↑ SOD ↓ CAT ↓ GSH-Px ↓	[83]
4	Kunming mice	Not reported	1000 mg/kg daily subcutaneous injection	6 weeks	22 months	Collagen ↓ Dermal thickness ↓ Collagen fibers are shorter, less compact, and more disordered Type I collagen fibers ↓ Type III collagen fibers ↑	[68]
5	Kunming mice	Male	400 mg/kg intraperitoneally	30 days	8 weeks	Destruction of hair follicles in skin Skin HYP ↓	[69]
6	Kunming mice	Male and female	1000 mg/kg daily subcutaneous injection	42 days	6 weeks	SOD ↓ MDA ↑ Skin HYP ↓ Elastin ↓ Skin moisture ↓ Dermal thickness ↓ Collagen ↓ HYP ↓	[70]
7	Kunming mice	Male	150 mg/kg daily intraperitoneally	6 weeks	Unknown	Dermal thickness ↓ HYP ↓	[71]
8	Kunming mice	Female	1000 mg/kg daily subcutaneous injection	42 days	3 months	MDA ↑ and GSH-Px ↑ HYP ↓	[72]
9	Kunming mice	Female	1000 mg/kg daily subcutaneous injection	30 days	8 weeks	CAT ↓ SOD ↓ GPH-Px ↓ HYP ↓ MDA ↑	[73]
10	Kunming mice	Male	200 mg/kg daily subcutaneous injection	8 weeks	6 weeks	H_2_O_2_ ↑ MDA ↑ CAT ↓ GSH ↓ GSH-Px ↓ Dermal thickness ↓ Skin integrity and hair follicles were also impaired Collagen ↓ Skin collagen fiber content is reticular, more loose, and irregular	[84]
11	Kunming mice	Male	500 mg/kg daily intraperitoneally	8 weeks	Unknown	Skin moisture ↓ HYP ↓ Dermal thickness ↓ Collagen ↓ SOD ↓ MDA ↑ CAT ↑ and GSH-Px ↑ p16 ↑ and p21 ↑ Sirtuin1 ↓ and cyclin D1 ↓	[74]
12	Kunming mice	Male	200 mg/kg daily intraperitoneally	30 days	Unknown	HYP ↓	[75]
13	Nude mice	Not reported	1000 mg/kg daily subcutaneous injection	8 weeks	6 weeks	Skin elasticity ↓ Dermal thickness ↓ SOD ↓ MDA ↑ AGEs ↑ CD31 expression ↓.	[76]
14	Nude mice	Male	1000 mg/kg daily subcutaneous injection	3 weeks	6 weeks	Collagen ↓ Dermal thickness ↓	[77]
15	Nude mice	Not reported	1000 mg/kg daily subcutaneous injection	8 weeks	6 weeks	AGE ↑ SOD ↓ MDA ↑ Dermal thickness ↓ Collagen ↓	[78]
16	BALB/c	Male	500 mg/kg daily per oral administration	6 weeks	12 weeks	Fibroblast count ↓ Collagen ↓	[86]
Rats							
17	Wistar	Male	125 mg/kg daily subcutaneous injection	6 weeks	Unknown	Dermal thickness ↓ Skin moisture-no difference HYP ↓ Collagen type I expression ↓	[79]
18	Wistar	Female	150 mg/kg daily intraperitoneally	12 weeks	170-180 days	Skin moisture ↓ HYP ↓	[80]
19	Wistar	Male	200 mg/kg daily intraperitoneally	6 weeks	Unknown	Rats had brittle and less elastic hair Thin, inelastic, and sagged skin is reported	[85]
20	Sprague-Dawley	Male	1000 mg/kg daily subcutaneous injection	8 weeks	4-6 weeks	Skin elasticity ↓	[81]
21	Sprague-Dawley	Male and female	100 mg/kg daily hypodermically injected	8 weeks	Unknown	CAT ↓ and GSH-Px ↓ MDA ↑ Dermal thickness ↓.	[82]

HYP: skin hydroxyproline; SOD: skin superoxide dismutase; MDA: malondialdehyde; GSH-Px: glutathione peroxidase; CAT: catalase; GSH: glutathione S-transferase.
